# Finger Movement Recognition via High-Density Electromyography of Intrinsic and Extrinsic Hand Muscles

**DOI:** 10.1038/s41597-022-01484-2

**Published:** 2022-06-29

**Authors:** Xuhui Hu, Aiguo Song, Jianzhi Wang, Hong Zeng, Wentao Wei

**Affiliations:** 1 State Key Laboratory of Bioelectronics, Nanjing, China; 2 Jiangsu Key Laboratory of Remote Measurement and Control, Nanjing, China; 3 School of Instrument Science and Engineering, Southeast University, Nanjing, China; 4 School of Design Arts and Media, Nanjing University of Science and Technology, Nanjing, China

**Keywords:** Biomedical engineering, Electromyography - EMG, Motor neuron

## Abstract

Surface electromyography (sEMG) is commonly used to observe the motor neuronal activity within muscle fibers. However, decoding dexterous body movements from sEMG signals is still quite challenging. In this paper, we present a high-density sEMG (HD-sEMG) signal database that comprises simultaneously recorded sEMG signals of intrinsic and extrinsic hand muscles. Specifically, twenty able-bodied participants performed 12 finger movements under two paces and three arm postures. HD-sEMG signals were recorded with a 64-channel high-density grid placed on the back of hand and an 8-channel armband around the forearm. Also, a data-glove was used to record the finger joint angles. Synchronisation and reproducibility of the data collection from the HD-sEMG and glove sensors were ensured. The collected data samples were further employed for automated recognition of dexterous finger movements. The introduced dataset offers a new perspective to study the synergy between the intrinsic and extrinsic hand muscles during dynamic finger movements. As this dataset was collected from multiple participants, it also provides a resource for exploring generalized models for finger movement decoding.

## Abstract (summary)

**Table:** 

Measurement(s)	Electromyography
Technology Type(s)	Surface Electrode
Factor Type(s)	hand gesture
Sample Characteristic - Organism	Homo sapiens
Sample Characteristic - Environment	human house
Sample Characteristic - Location	China

## Background & Summary

The motor neuronal activity within muscle fibres has been widely observed through surface electromyography (sEMG). The sEMG-based myoelectric control systems have been extensively exploited in the fields of rehabilitation engineering and robotics (especially for the design of bionic prosthetics^1–3^ and exoskeleton devices^4,5^). Recently, high-performance myoelectric systems have been sought for accurate identification of finger movements and forces^6–9^. Such systems would enable more intuitive and streamlined human-computer interactions (HCI) in modern applications of surgical robotics^10^, tele-operated robotics^11,12^, or virtual reality environments^13,14^. However, as the sEMG signal is measured at the skin surface, the source signals produced by motor neurons not only undergo low-frequency filtering caused by muscle, fat and subcutaneous tissues, but they are also contaminated by elusive artefacts such as those associated with electromagnetic interference and electrode displacement^15^. Separating the source signals of the forearm sEMG signal is still quite challenging because of the complex spatial muscle composition^16^, and hence this complicates the process of decoding dexterous finger movements from sEMG signals.

**Fig. 1:**
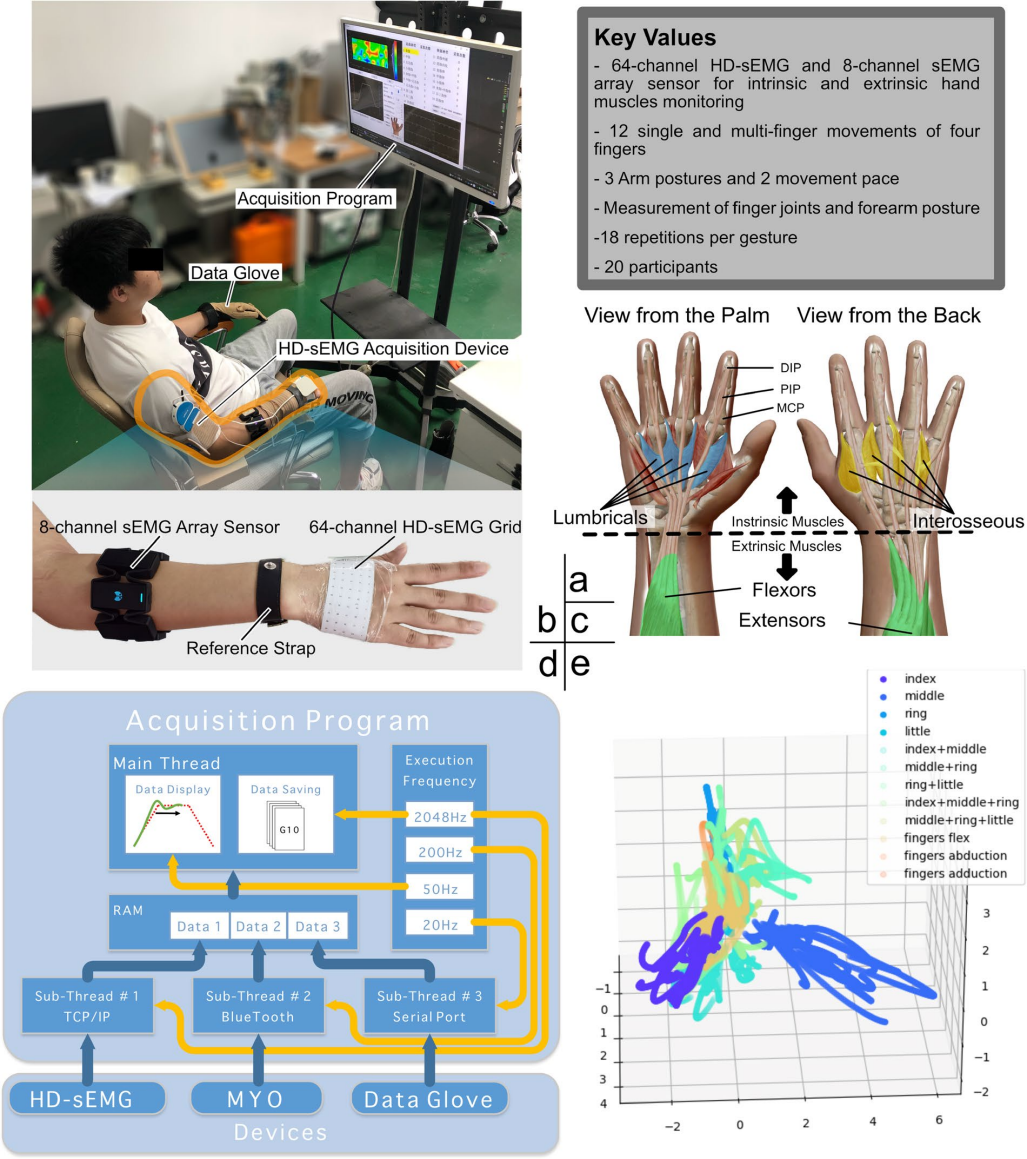
Experimental and analysing workflow for sEMG data acquisition and analysis. (**a**) Key experimental settings (**b**) The experimental setup and the sEMG sensor arrangement. (**c**) Hand muscle anatomy and the extrinsic and intrinsic hand muscles. (**d**) The data acquisition framework. (**e**) An example for sEMG data analysis. Principal component analysis (PCA) is adopted to capture and visualize the spatial distribution of the low-dimensional HD-sEMG features under different gestures. The PCA plot shows that the single finger movements are highly uncorrelated, while the multi-finger ones are strongly correlated.

**Fig. 2:**
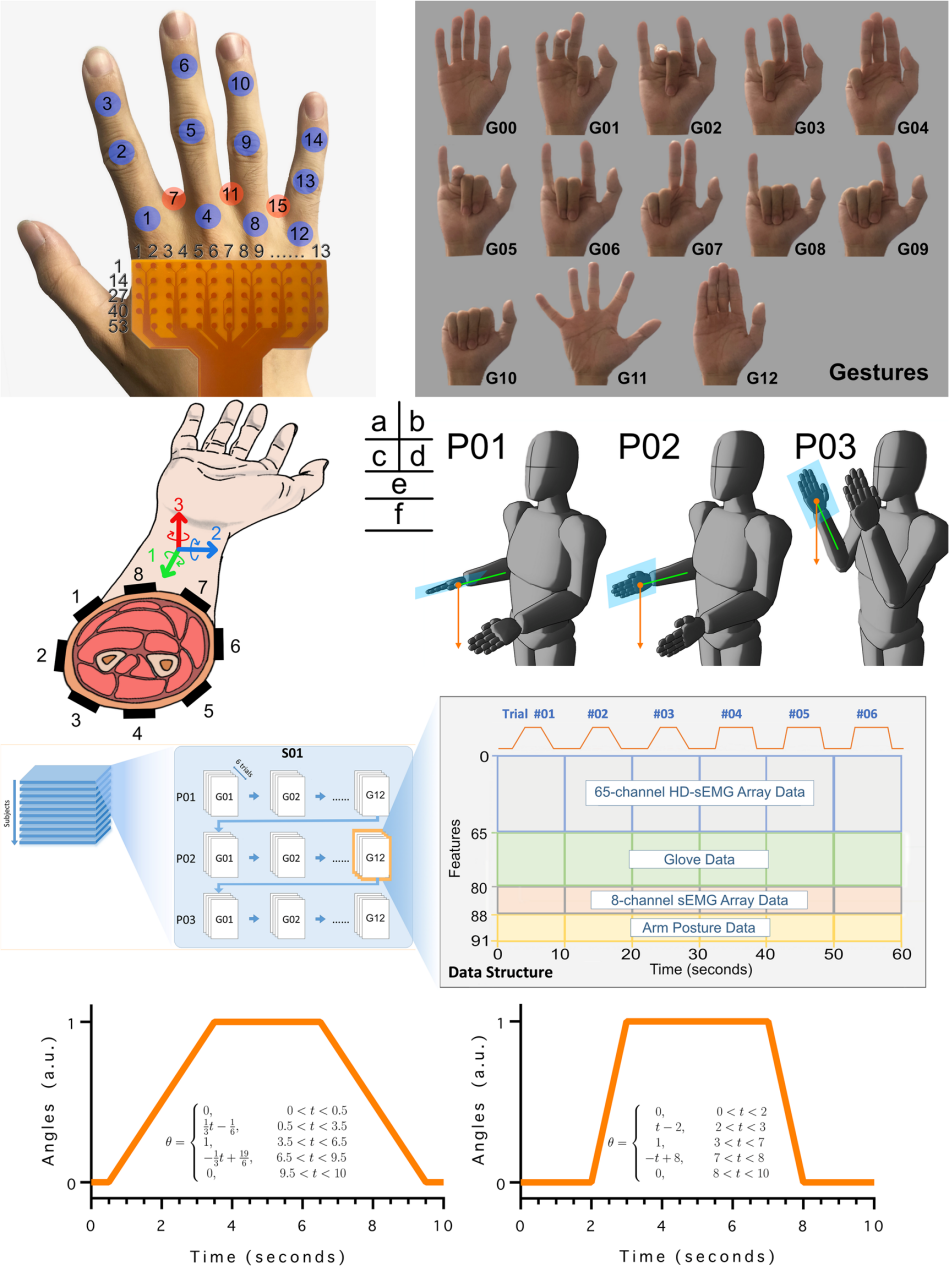
The structure and specification of the HD-sEMG database. (**a**) The feature indices of the HD-sEMG and data glove. (**b**) Images of the 12 hand gestures and the rest gesture G00. (**c**) The feature indices of the sEMG array data and the IMU data of the MYO armband. (**d**) Images of three arm postures. (**e**) The data structure. The arrows in the blue block denote the order of saving the data records. The right magnified graph denotes the data structure of one gesture. The horizontal and vertical axes represent the time and feature indices, respectively. (**f**) The finger motion trajectory in the target trajectory guidance experiment (TGE).

Recently, some studies of the myoelectric physiology suggested the presence of the sEMG sources in the intrinsic hand muscles as well as the external ones^17–19^. This is because the motor neurons of these intrinsic and extrinsic muscles share a common synaptic input during voluntary movements^17,20^, and this indicates a potential relevance between the associated sEMG sources. In this paper, we collected a multi-source sEMG dataset in order to investigate the relationship between unrestricted finger movements and the sEMG signals of hand muscles. Our approach stands out among the vast majority of the myoelectric control approaches that focus on the extrinsic hand muscles only^21–26^, while we simultaneously record signals from both the intrinsic and extrinsic hand muscles, as shown in Fig. 1(b). The signals of the intrinsic hand muscles were measured by a 64-channel high-density sEMG (HD-sEMG) grid^27^, which also allows the observation of the discharge patterns of the motor-unit action-potential trains (MUAPt)^28^. The signals of the extrinsic hand muscles were measured by a wearable 8-channel sEMG sensor array, which is commonly adopted by most of the pertinent commercial applications^26,27^. In addition, a data-glove sensor was used to measure the finger joint angles and synchronize them with the sEMG measurements. The collected data is essentially used to for automatic decoding of human finger movements based on myoelectric signals. In particular, 12 single-finger and multi-finger flexion movements of four fingers were examined, along with the abduction (moving away from the hand center) and adduction (moving towards the hand center) of the metacarpal joints. Images of 12 gestures are shown in Fig. 2(b). Due to the constraints associated with the measurement technique and experimental setup designed in this study, we focus on analysing these 12 movements for the four long fingers only. Fortunately, as the thumb has distinctive anatomical and functional characteristics, we were able to collect and analyse data from the four long fingers in a systematic manner. We established a sound data base for the analysis of the simultaneous movements of the four fingers, while maintaining the integrity of the study.

While the intrinsic hand muscles control only the metacarpal finger joints, the extrinsic hand muscles control the wrist as well as the proximal/distal interphalangeal finger joints^29^, as shown in Fig. 1(c). To minimize the muscle work, our body subconsciously keeps the forearm muscles into a resting length-tension state, where the wrist joint resists the gravity to maintain a neutral position^30^. Therefore, when fingers are moved, the forearm sEMG signals may arise from two types of source signals: signals that control fingers, and signals that maintain wrist posture. However, the majority of the sEMG databases and EMG-based hand gesture prediction approaches ignore the gravity effects. Therefore, we collected sEMG data under three typical arm postures in order to quantify the gravity effects on the wrist joints. In addition, we experimented with two finger movement speeds to better represent real myoelectric control scenarios. Moreover, the sEMG signal repeatability is commonly lower than that of the finger joint angles. Thus, we designed here three inter-session experiments, with each session containing 6 repeated trials (with three fast and three slow movements), with a total of 18 trials per gesture. This increases the abundance and diversity of the sEMG data.

We experimentally tested the finger movement repeatability and the synchronization of the multi-source data. Furthermore, we extracted low-dimensional components of the HD-sEMG activation map in order to visualize the relation between the single-finger and multi-finger movements. Finally, a baseline pattern recognition method was adopted to show the feasibility of dexterous finger movement classification based on the HD-sEMG signals of the intrinsic hand muscles. Our work is highly reproducible. In particular, twenty inexperienced participants were recruited for data acquisition setups that resemble real application scenarios, and enable the investigation of the robustness and generalization of EMG-based methods for identifying dexterous finger movements. Also, our dataset has 64 channels of HD-sEMG signals, and this high channel density enables the investigation of the MUAP discharge patterns of the intrinsic hand muscles. In general, our dataset paves the way of exploiting the signals of the intrinsic hand muscles for myoelectric control of dexterous finger movements.

## Methods

### Participants

We collected data from 20 volunteers who had no known skeletal and/or neuromuscular disorders (11 males and 9 females, with an age range of 24 ± 2 years, a palm circumference range of 19.4 ± 1.6 *cm*, and a forearm circumference range of 23.3 ± 2.8 *cm*). All participants were right-handed had no previous experience with myoelectric control or data-glove experiments. The demographic data of each participant is reported in the dataset^31^. The participants were recruited from the Southeast University in China via a mass email communication with a basic description of the experimental setup and goals. Each respondent who expressed interest was sent further details, and then scheduled to have a data acquisition session. All participants were given a full verbal description of the methods, purposes, and protocols for the experiments. Each participant read and signed an informed consent form before proceeding with the experiments. The experimental protocol was approved by the Ethics Committee of Jiangsu Province Hospital (2020-SR-362). All experiments were thus conducted according to the ethical guidelines for medical research involving human participants (according to the Declaration of Helsinki^32^).

For our database, the participant count and the selection criteria were based on two aspects. Firstly, we examined 14 publicly available sEMG databases, and found that they have mean and median numbers of participants of 20.42 and 20 (the list of the examined databases can be found in our dataset repository^31^), respectively. Secondly, we considered the abundance of the sEMG features in the existing datasets. Indeed, Atzori *et al*.^33^ demonstrated that the EMG classification accuracy had negative correlation with the body mass index (BMI) (p = 0.026), but no significant correlation with gender (p = 1), hand dominance (p = 0.921) or age (p = 0.117). This means that a population spanning a wider range of BMI values may have more abundant sEMG features. Therefore, we selected participants with diverse BMI values ranging from 16.0 to 29.74, including “moderately thin” to “pre-obese” individuals according to the WHO standard for South-East Asia.

### Data acquisition setup

The HD-sEMG has the advantage of revealing the muscular activation patterns with a high spatial resolution, and the flexible HD-sEMG electrode grid is suitable for sEMG acquisition on a non-flat surface (e.g., the back of a human hand). The 64-channel HD-sEMG electrode grid (OT Bioelectronica, Italy) used here is made from a 0.1 mm flexible printed-circuit film. The 64 electrodes were organized into 5 rows × 13 columns (with one void corner spot), where each electrode is circular with a diameter of 3 mm, and an 8 mm centre-to-centre distance. The electrode grid covers a surface area of 105 × 40 *mm*^2^, and it was used to record the sEMG signals on the back of the hand, as shown in Fig. 1(b). The HD-sEMG signals were acquired and amplified using a portable sEMG amplifier (Sessantaquattro, OT Bioelectronica, Italy) through wireless *TCP*/*IP* communication (shown in Fig. 1(d)). A reference electrode band was strapped around the wrist bone. The HD-sEMG signals were detected in a monopolar mode, with a sampling rate of 2048 Hz and an A/D resolution of 16 bits. A multichannel simultaneous sampling chip (ADS1298, Texas Instruments) was used with a common mode rejection ratio (CMRR) of 115 dB and a gain bandwidth product (GBP) of 100 kHz. To ensure a low electrode-skin contact impedance, we used an abrasive paste (Nuprep, Weave Ltd.) to rub the skin prior to electrode placement, and then brushed a conductive paste (Ten20, Weave Ltd.) on each grid with a 1-mm thickness. Moreover, unrestricted finger movements may be accompanied by changes in the bony arch, causing the electrodes to peel off and get detached from the back of the hand. Therefore, we fixed the palm shape and prevented hand skin deformation using a customized thermoplastic socket.

The sEMG signals of the forearm muscles were recorded (at a sampling rate of 200 Hz and an A/D resolution of 8 bits) by a MYO armband (Thalmic Labs, Canada), which is a commercial myoelectric armband with eight bipolar dry-electrode channels. This MYO armband device uses high accuracy zero drift micropower operational amplifiers (TSZ124, STMicroelectronics) with a CMRR of 115 dB and a GBP of 400 kHz. Recently, Pizzolato *et al*.^34^ compared the gesture classification accuracies for four types of sEMG acquisition devices (including the Delsys Trigno electrode and the MYO armband), and they reported comparable results for the Delsys Trigno electrode and the MYO armband. This indicates that the relatively low sampling rate and number of channels of the MYO armband will not have a significant impact on the gesture classification accuracy. In addition, several independent studies^35–37^ have also demonstrated the effectiveness of the MYO armband in gesture recognition applications. So, the MYO data should be reusable and valuable in sEMG-based HCI applications. The sEMG and IMU data of MYO armband are transferred via bluetooth communication protocol (shown in Fig. 1(d)). In addition, arm posture information was extracted by the inertial measurement unit (IMU) of the armband. The armband and the HD-sEMG grid were worn on the same side of the right hand. Compared with the HD-sEMG, this circular arrangement is easy to setup, and has been adopted earlier for numerous databases^21,37,38^.

The positioning of the HD-sEMG grid is shown in Fig. 2(a), where the first column of the grid is aligned with the 2nd metacarpal, and the signals associated with the 1st to the 4th dorsal interosseous muscles are measured in this setup. For some of the participants whose hands were small compared to the HD-sEMG array, some of the hypothenar group muscles were partially covered, including the abductor digiti minimi muscle and the flexor digiti brevis. Also, for participants whose forearm circumferences are smaller than the inner circumference of the MYO armband, an elastic band is wrapped around the MYO armband to ensure good electrode-skin contact. For each participant, the specific positioning information of the HD-sEMG grid and the MYO armband was recorded in the collected dataset^31^.

Finger kinematics were captured using a 22-sensor data-glove (CyberGlove II, Cyber Glove Systems LLC) whose data is transferred to a computer via a wired serial communication protocol (shown in Fig. 1(d)). The recorded kinematic data contained three bend movements per finger, and three abduction movements on the adjacent four fingers. As the finger flexion frequency in the experiments was lower than 0.5 Hz, the data-glove sampling frequency was set at 20 Hz to safely meet the Nyquist–Shannon sampling theorem. Compared to other computer-vision-based methods for finger joint recognition, our method is less influenced by finger occlusion and has higher accuracy of joint angle measurement. To synchronize the angle measurements with the sEMG signal acquisition, each participant put on the glove on the opposite side of the sEMG acquisition device, and conducted corresponding finger movements during the acquisition process.

The experimental setup is shown in Fig. 1(b). Each participant was comfortably seated on a chair with a straight back and relaxed shoulders in front of a 40-inch LCD monitor on which the experiment information was displayed. This information included stimuli (the finger movements the participant needs to make) and multiple types of relevant data (such as the finger joint angles, the HD-sEMG activation map, and the sEMG data of the MYO armband). The data acquisition was controlled by a laptop through a self-developed Qt Program. The acquisition workflow is shown in Fig. 1(d), where the three acquisition devices had independent threads, and batches of data from the three devices were synchronized and fetched from the random access memory (RAM) of the computer. The raw data acquisition was conducted using a PC running 64-bit Microsoft Windows 10 with an Intel i7 1.73-GHz processor, and an 8-GB RAM.

### Experimental protocol

The data-glove output was initially calibrated to normalize the glove analogue signals in terms of absolute degrees. Prior to data collection, the maximum voluntary contraction (MVC) force of each movement was measured during isometric contraction (i.e. fingers being flexed to the maximum degree), and participants were asked to learn to exert 70% of the MVC for each gesture. In order to prevent muscle fatigue associated with maximal finger joint flexion during the data collecting, the participants were instructed to exert about 70% of the MVC according to their training memory. The dataset structure is described in Fig. 2(e). Each experiment had three sessions, and in each session, a participant was required to complete three fast and three slow trials in turn under different arm postures (Fig. 2(d)). For each session, data acquisition lasted around 40 minutes (not including breaks).

To ensure the consistency of the finger motion speed, and the start/stop times, we designed a target trajectory guidance experiment (TGE). In particular, the joint angle curves and the target trajectories were displayed on the monitor in real-time during data collection (e.g., See “Data Display” in Fig. 1(d)). The participants need to control their finger joints to track these trajectories.

During data acquisition for different gestures, the MCP joint data of one finger was selected for target tracking. For multi-finger movements, the data of only one finger is displayed in order to avoid visual confusion. Table 2 shows the specific joint selections for each of the 12 gestures. Also, detailed descriptions of the finger movements and the arm postures are shown in Fig. 2(b) and Fig. 2(d), respectively. The fast and slow finger movement trajectories are shown in Fig. 2(f). The acquisition time of each trial is 10 seconds, and the data collection is automatically stopped when time is up. A trial can be accepted or rejected based on observations of a participant’s tracking performance

Before initiating the data acquisition, the participants were allowed enough time to examine and learn tracking along the target curves. A trial was started once a participant was ready. Two researchers readily answered any questions a participant might have. Also, participants were instructed to always start and finish trials in the resting position (with the arm posture fixed and the fingers relaxed). Each participant had a break at the middle of each session (after the first 6 movements), and also was allowed to have rest at any other time. For experiments lasting over 50 minutes, each HD-sEMG sensor would be replaced to avoid conductive paste melting and increase the crosstalk between the electrodes.

### Data processing

The HD-sEMG data was filtered through a zero-phase 3^rd^ order band-pass Butterworth filter with lower and upper cut-off frequencies of 10 and 500 Hz, respectively. Since the original 13 × 5 electrode grid had one electrode missing at one corner, the signal value at that location was estimated as the average of the values from three adjacent grids. The instantaneous signal could thus be reshaped as an image. Finally, the units for both the HD-sEMG and the MYO armband signals were set to be millivolts.

For data validation, the sEMG signals were pre-processed and transformed into envelope signals. This pre-processing procedure was packed as an easily reusable code module for both offline and online processing. Firstly, notch filtering at 50.5 52 Hz was performed to eliminate power-line interference. Then, an outlier detection algorithm was used to screen out any abnormal signals that may result from poor electrode-skin contact. Here, we applied the Pauta criterion for outlier detection in HD-sEMG signals. Specifically, because the instantaneous sEMG amplitude can be modelled by a Gaussian distribution^39^, we set the probability of a normal sEMG as 99.7%. Then, the instantaneous amplitude of HD-sEMG outside the *μ* ± 3*σ* range were considered as outliners. Each outlier signal was replaced by the average of the remaining normal signals. The details of these processing steps are shown in Table 1. Indeed, conventional rectification and calculation of the root-mean-square (RMS) value for 400 ms intervals were performed to obtain low-frequency sEMG activation signals.

**Table 1:** Data processing pipeline.

	Step	Function
Data Saving	1	Zero-phase 3^*rd*^ order band-pass Butterworth filter [10Hz500Hz]
	2	Patching the missing electrode of the HD-sEMG grid
	3	Unit transform (“Data_1” and “Data_2” is referred to Fig. 1(d)): HD-sEMG signals: Data_1 × 0.000286 (mV); MYO signals = Data_2 × 0.04 (mV);
	4	Solving the Euler angles from the quaternion data of MYO armband
Data Validation	5	Zero-phase 3^*rd*^ order band-stop Butterworth filter [50.5Hz52Hz]
	6	Outlier Detection
		(1) Screen out the signal that outside *μ* ± 3*σ* according to the empirical rule, then replace the original signal with the average of the remaining normal signals.
		(2) Repeatedly run step (1) until the no outliers is detected
	7	Rectification, get the absolute value of the sEMG
	8	Computing root mean square value from intervals of 400 ms duration

**Table 2:** Movement instructions for the upper limbs.

	Instruction
Arm	P01: The elbow is bent at a 90 degree, the palm surface is parallel to the ground.
	P02: The elbow is bent at a 90 degree, the palm surface is vertical to the ground.
	P03: The elbow is bent at a 45 degree, the palm surface is vertical to the ground.
Hand	(G01~G04): Single finger flexion: Bring your index/middle/ring/little finger towards the palm of your hand, then return to the resting position (G00).
	(G05~G07): Two adjacent fingers flexion: Bring your index and middle, or middle and ring, or ring and little fingers towards the palm of your hand, then return to G00.
	(G08~G09): Three adjacent fingers flexion: Bring your index, middle and ring fingers, or middle, ring and little fingers towards the palm of your hand, then return to G00.
	(G10): Bring your four fingers towards the palm of your hand, then return to G00
	(G11~G12): Four fingers abduction and adduction
	The selection of finger joint data for TGE: Choosing one of the MCP joint in the current gesture, the priority of the selection is: 1) Index (No. 1); 2) Middle (No. 4); 3) Ring(No.8); 4) Little(No.12); 5) Abduction(No.15) The numbers in parentheses are the indices (1–15) of the finger joint data marked in Fig. 2(a). For instance, No. 4 was selected in G06 to track the target curve, and No.1 is selected in G08

## Data Records

The data collected in our work (as well as the employed data collection methods) can be found at figshare^31^. Specifically, our data is organized according to the following file structure:

• Info.xls

• S01

– *S01.cal*

– *S01.png*

– G01.mat

– G02.mat

……

– G12.mat

• S02

……

• S20

In particular, a Microsoft Excel file (‘Info.xls’) contains essential pieces of information for each participant (including age, gender, hand size, experimental remarks, etc.). The data files of the 20 participants are stored in ‘S01’ through ‘S20’, respectively. Each participant folder (e.g. ‘S01’) contains 14 files, among which ‘S01.cal’ is the data-glove calibration file; ‘S01.png’ is the experiment image; and the other 12 ‘Gxx.mat’ files are the collected data (where xx represents an index for each of the 12 gestures).

To illustrate the data storage structure, we take ‘G01.mat’ as an example. All data is stored into a two-dimensional matrix within the struct variable ‘Data’. The 91 matrix columns represent the data features: the HD-sEMG features are those of columns 1–65; the glove features are those of columns 66–80; the MYO armband features are those of columns 81–88; and the arm posture features are those of columns 89–91. The feature index is shown in Fig. 2(a,c). The rows of the data matrix represent the temporal axis, and the data from three experiment sessions are saved in order of occurrence. Figure 2(d) shows the storage order for one session. Each session contains 6 repeated trials, namely three slow trials and three fast trials. The temporal length of each trial is 10 seconds, and the data sampling rate is 2048 Hz. So, the data matrix has 368640 rows (corresponding to 3 sessions × 6 repetitions × 10 seconds × 2048 Hz). In conjunction with the ‘Sxx’ folders, the ‘Code’ folder includes Jupyter Notebook scripts that generate some exploratory plots for ‘Data Validation.’

In order to quantify the gravity effects on the wrist joint, we collected sEMG data under three typical arm postures, which are denoted as P01, P02, P03 as shown in Fig. 2(e). As the participants performed the 12 finger movements, they were asked to maintain these three postures over three sessions, respectively. During data acquisition, a participant can keep the shoulder relaxed and keep the wrist in a neutral position, but should leave the forearm unsupported. The 12 considered finger movements are shown in Fig. 2(b). In each session, the participant was instructed to make these finger movements in order (1–12), where each movement was repeated 6 times.

## Technical Validation

**Fig. 3:**
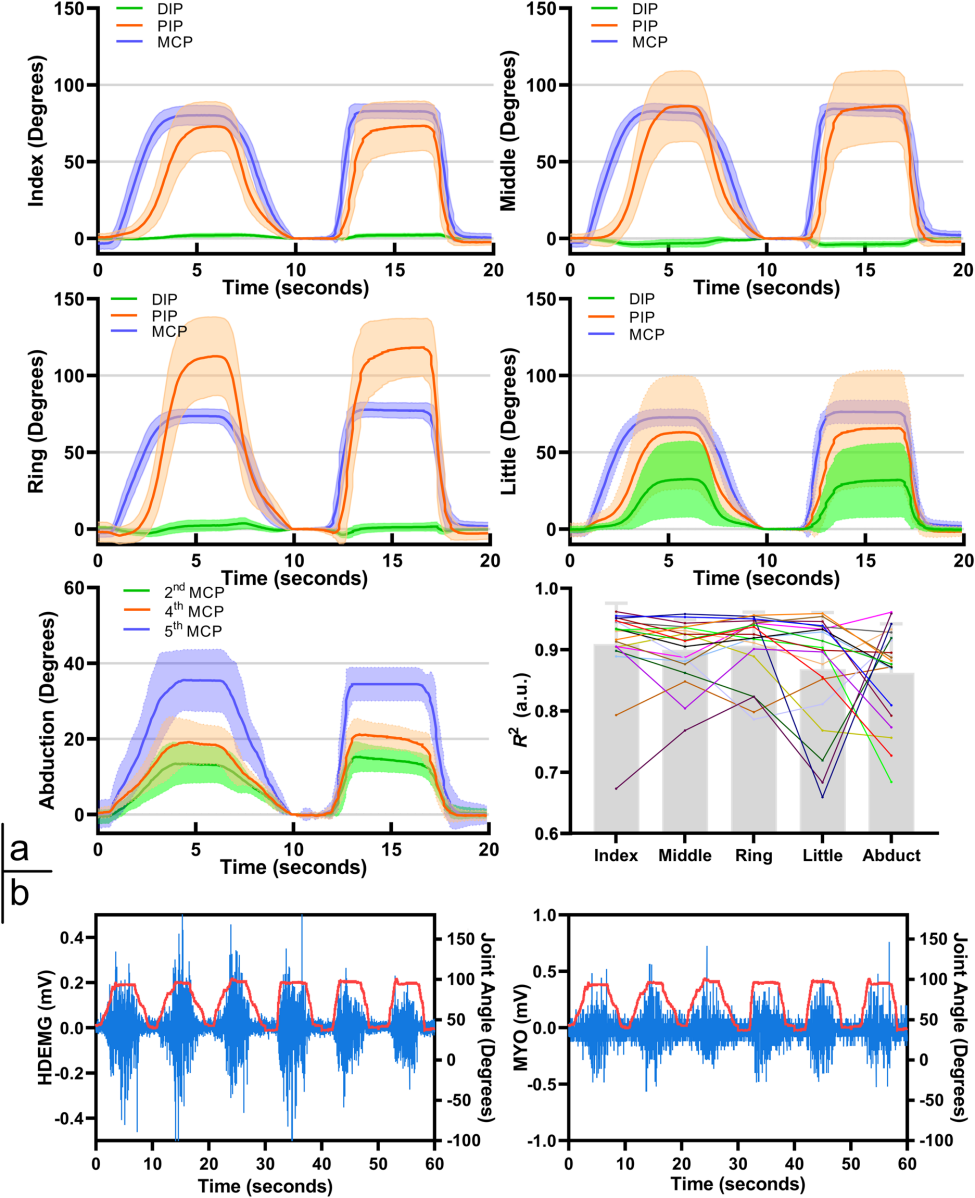
Results of the data repeatability and synchronisation experiments. (**a**) The trajectories of flexion and abduction of the metacarpal joints of the four long fingers with error bands. The bottom-right statistical bar plots denote the average coefficient of determination $\overline{{R}^{2}}$ between the true motion trajectory and the target trajectory. (**b**) Synchronisation between the sEMG and data-glove signals.

Repeatability Data repeatability is crucial for successful EMG-based myoelectric control systems. We examined the data repeatability as follows. We calculated the coefficient of determination (*R*^2^) between the target motion curve and the finger joint angles on a sequence of one fast trial and one slow trial (with a total of 20 seconds), as shown at the bottom right of Fig. 3(a). For each of five joints (index, middle, ring, little and metacarpal abduction), calculations were made at least 9 times (3 sessions × 3 repetitions). As some joints (such as the index and middle joints) are frequently used for target curve tracking in the TGE, the *R*^2^ coefficient was evaluated on more sequences associated with those movement joints. Then, we calculated the average coefficient of determination ($\overline{{R}^{2}}$) for each joint. The statistical bar plots show that the $\overline{{R}^{2}}$ values for the five joints are above 0.85, and this indicates good repeatability. The polylines show the $\overline{{R}^{2}}$ value of the 20 participants for each joint. For the adduction movement (G12), it is difficult to always return to the same initial position. This means that the repeatability of the metacarpal movement is lower than those of the other joints. In addition, a few participants demonstrate lower repeatability on their little fingers.

Figure 3(a) also shows the motion curves for all finger joints of a representative participant (S01). The results show that the MCP joint movement precedes those of the PIP and DIP joints, and the DIP joint moves less than the other joints. Indeed, these results also support the necessity of using the MCP joints for trajectory tracking. Moreover, the TGE results show that the participants can uniformly perform slow and fast trials, and that the error band width is acceptable.

### Synchronisation

The data acquisition process ensures accurate mapping between the sEMG signals and the finger joint angles. Figure 3(b) shows two sEMG signals (associated with a certain HD-sEMG channel and the MYO armband) on the same time axis, where the red curve represents the glove data of one of the finger joints in association with a specific gesture.

### Classification

To evaluate the baseline performance of finger movement recognition, we applied a simple K-nearest neighbour (KNN) classifier (with K = 15)^40^. The 12-finger-movement KNN classification performance was individually evaluated for each participant. To achieve this, we firstly pre-processed the HD-sEMG and the MYO signals as input features as described in Table 1. Then, the gesture number (1–12) was used as the data label for myoelectric signal classification. For each gesture, we randomly selected 13 out of 18 data trials (72.2%) as the training set, while the remaining 5 trials (27.8%) were used for testing. This data splitting scheme shall prevent model overfitting, because the intra-trial samples (i.e. samples within the same repetition trial) are strongly correlated, a total shuffling scheme across the 18 trials may cause the data to leak from the training set to the test one^41^.

To compare the prediction accuracy associated with the extrinsic hand muscles against that of the intrinsic hand muscles, we used the instantaneous RMS of the HD-sEMG and MYO signals separately to train two KNN models (namely, the “HDEMG” group and the “MYO” group). In addition, we designed a “FUSION” group which combines the HD-sEMG and MYO features. The values of the two feature types are respectively scaled into the range of [0, 1]. All HD-sEMG channels share the same scale factor, while all the MYO channels have a different scale factor. Subsequently, we combined the features of the two types into a new feature vector (of 73 dimensions) and conducted KNN classification.

**Fig. 4:**
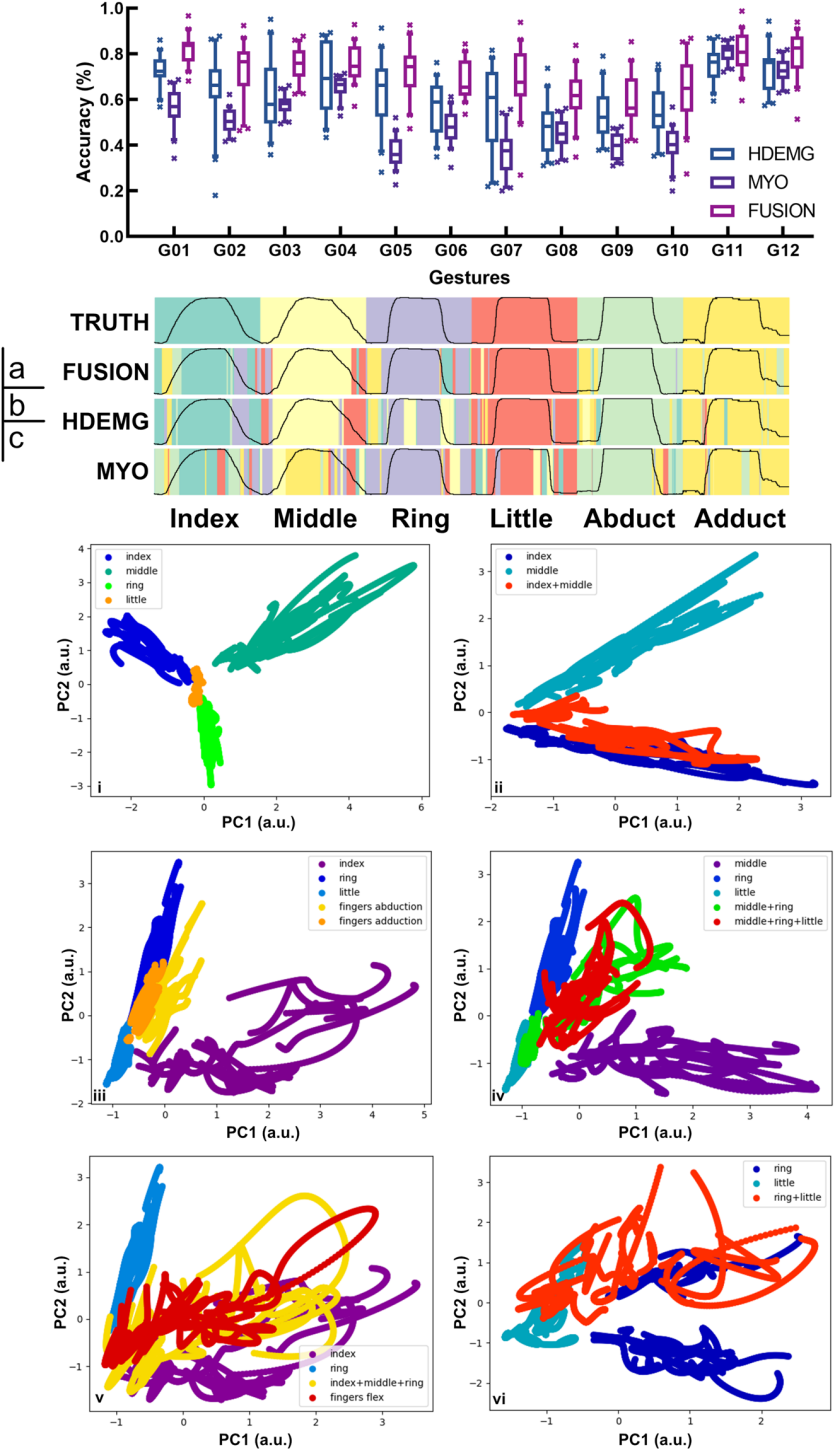
Classification results of the finger movements based on the HD-sEMG signals. (**a**) Classification accuracies of 12 finger movements. (**b**) Online prediction of 6 single-DOF finger movements. (**c**) Two-dimensional principal components for the 12 finger movements.

Statistical box plots of the predicted accuracies averaged over the 20 participants are shown in Fig. 4(a). The statistical results show that the mean classification accuracy for the 12 gestures in the FUSION group (71.82 ± 7.57%) is higher than that of the HDEMG group (62.48 ± 8.82%), which, in turn, is higher than that of the MYO group (52.56 ± 14.39%). Also, the prediction accuracies of single-finger movements and metacarpal movement (i.e. abduction and adduction) are higher than those of multi-finger movements. In addition, the standard deviation of the prediction accuracy associated with the intrinsic hand muscles is greater than that of the extrinsic hand muscles. This is possibly due to differences in the movement patterns among the participants. Moreover, some participants were found to have weaker HD-sEMG signals than the MYO signals during motion, even though the exerted forces reached their MVC values. Most likely, those participants tend to mainly control the PIP and DIP joints (via extrinsic wrist flexors) during flexion, while the other participants can simultaneously control three finger joints by exploiting both their intrinsic and extrinsic muscles. Figure 4(b) shows the online classification results of six single degree-of-freedom (DOF) movements (G01-G04, G11 and G12) for one representative participant (S07). The black curve represents the finger joint data. Also, the true labels, and the predicted labels from the FUSION, HDEMG, and the MYO groups are vertically arranged.

### Dimensionality reduction analysis

The principal component analysis (PCA) of the HD-sEMG signals offers a low dimensional visual perspective on the relationship between dexterous finger gestures. We considered HD-sEMG signals of the steady-state movement, i.e. the middle 3-second segment of every 10-second trial. Then, the 65-dimensional HD-sEMG signals (including patched corner signals) were reduced into 3-dimensional signals whose components can be plot as shown in Fig. 1(e) for a sample distribution of the afore-mentioned representative participant (S07). Obviously, the clusters of the index finger, the middle finger and the ring finger are spatially vertical to each other. This indicates linear independence among the movements of the three fingers. As well, the amplitude associated with the little finger is smaller than those of the other fingers, and the little finger data is also highly correlated with that of the ring finger in this 3D feature space.

To better visualize the association among the multi-finger movements, we selectively present principal components of specific finger movements in the 2D plane. Figure 4(c) shows the 2D principal component analysis results of Fig. 1(e). These results show closer correlation between data samples collected from the same trial. So, multiple curve segments (rather than highly discrete sample points) are presented in the low-dimensional feature space. Figure 4(c-i) show the 2D spatial distributions of the single movements of four fingers (other than the thumb). Clearly, the steady-state movement patterns of these four fingers are distributed in different spatial regions. This justifies the high motion prediction accuracy for single-DOF gestures. Figure 4(c-ii) through Fig. 4(c-vi) show the association between the multi-finger movements and the corresponding single-finger movements. The results show high feature aliasing among similar multi-finger movements. In addition, Fig. 4(c-vi) shows two centroids in the cluster of the ring finger, and this indicates that the features have shifted across sessions.

### Signal quality

Prior to data collection, the signal quality of HD-sEMG was assessed by measuring the electrode-skin impedance in the OTBioLab+ Software (v1.5.6, OT Bioelettronica), with an average impedance below 40 *kΩ*. During data acquisition, the EMG artefacts were continually monitored. In offline analysis, the signal frequency spectra were checked through frequency analysis. As the sEMG intensity varies between different gestures, different signal-to-noise ratios (SNR) are obtained. Vecchio *et al*.^27^ concluded empirically that, at low EMG amplitudes, the noise power should be no more than one half of the signal power to ensure reliable decomposition. The statistical results show that the average SNR of each gesture of HD-EMG is 8.76 ± 1.26 *dB*, and the average SNR of each gesture of MYO is 20.60 ± 6.20 *dB*.

## Usage Notes

Two types of electrodes were used in the experiments, namely ELSCH064NM3 and GR08MM1305. Some participants used both types of electrodes during the three sessions. There is indeed no difference between the two types of electrodes, except for the position of the missing electrode. The electrode type used by each participant is specified in the collected dataset. Experimental videos of all participants are available from the authors upon request.

The A/D conversion coefficient of HD-sEMG is officially calibrated, and the unit of HD-EMG signal in this database has been converted into millivolts. However, there is no official coefficient for MYO, the coefficients in Table 1 was measured under the laboratory conditions, so the MYO data of this database is the digital signal. The unit conversion of MYO was completed in the pre-processing program, which can be in the “OfflineAnalysis.ipynb” script of “Code” folder of our figshare project^31^.

All the participants are able to complete the 12 types of movements, as they are commonly used in the daily interaction, but we did find some of the fingers (mainly for the ring finger) can involuntarily follow the other active fingers. Therefore, we allow this involuntary following movement happen, as long as only the fingers within the predefined gestures are correctly activated.

There are also some of the limitations that should be discussed: First, in order to minimize the impedance of electrodes, the preparation for HD-sEMG signal collection is a bit tedious at present, so the electrode material optimization may help improve the ease of wearing. Second, this dataset does not measure the hand force due to the limitations on experimental equipment. Our future works include extending the current intrinsic hand muscles recording to hypothenar group muscles, and developing stretchable electrode materials to improve the robustness against electrode displacement. Furthermore, we will expand the contents of this dataset toward more hand motion scenarios, such as hand manipulation tasks, which helps to decode the hand motion intentions during the interaction between human and environment.

## Data Availability

The custom Jupyter Notebook scripts for some exploratory plots in Technical Validation section can be found in the “Code” folder of our figshare repository^31^. The acquisition program can be found in https://github.com/xuhui-hu/SciData2021.
